# Rationale and design of Children’s Oncology Group (COG) study ACCL20N1CD: financial distress during treatment of acute lymphoblastic leukemia in the United States

**DOI:** 10.1186/s12913-022-08201-0

**Published:** 2022-06-28

**Authors:** Melissa Beauchemin, Sheila Judge Santacroce, Kira Bona, Ha Dang, Sarah Alexander, Kamala Allen, Crystal De Los Santos, Beth Fisher, Yudy Muñeton-Castaño, Olivia Ponce, Sarah Vargas, Aaron Sugalski, Lillian Sung, Susan Parsons

**Affiliations:** 1 Columbia University School of Nursing, New York, NY USA; 2 Herbert Irving Comprehensive Cancer Center, Columbia University Irving Medical Center, New York, NY USA; 3 School of Nursing, University of North Carolina Chapel Hill, Chapel Hill, NC USA; 4 Lineberger Comprehensive Cancer Center, University of North Carolina, Chapel Hill, NC USA; 5 Department of Pediatric Oncology, Division of Hematology/Oncology, Boston Children’s Hospital, Harvard Medical School, Dana-Farber Cancer Institute, Boston, MB USA; 6 Department of Population and Public Health Sciences, Keck School of Medicine of USC, Los Angeles, CA USA; 7 Hospital for Sick Children, Toronto, ON USA; 8 Driscoll Children’s Hospital, Corpus Christi, TX USA; 9 Children’s Healthcare of Atlanta, Atlanta, GA USA; 10 Dana-Farber Cancer Institute, Boston, MA USA; 11 Children’s Oncology Group, Monrovia, CA USA; 12 University of Texas Health Science Center at San Antonio, San Antonio, TX USA; 13 Tufts Medical Center, Institute for Clinical Research and Health Policy Studies, Boston, MA USA

**Keywords:** Childhood all, Financial distress, Financial hardship, Financial toxicity, Health outcomes

## Abstract

**Background:**

The study purpose is to describe trajectories of financial distress for parents of children (ages 1–14.9 years) with newly diagnosed acute lymphoblastic leukemia (ALL). The secondary aim is to identify multilevel factors (child, parent, household, treating institution) that influence change in financial distress over time.

**Methods:**

The study uses a prospective cohort design, repeated measurements, and mixed methods. The settings are Children’s Oncology Group (COG) institutions participating in the National Cancer Institute Community Oncology Research Program (NCORP). Eligible participants are English- and/or Spanish-speaking parents or legal guardians (hereafter “parents”) of index children. Parents are asked to complete a survey during their child’s induction (T1) and maintenance therapy (T2), and near treatment completion (T3). Study surveys include items about (a) the child’s cancer and clinical course, (b) parental socio-economic status, financial distress and financial coping behaviors, and (c) household material hardships. At least 15 parents will be invited to participate in an optional semi-structured interview. NCORP institutions that enroll at least one parent must complete an annual survey about institution resources that could influence parental financial distress.

**Discussion:**

The results will inform future interventions to mitigate financial distress for parents of children diagnosed with ALL and could be instructive beyond this disease group.

**Trial registration:**

This trial was initially registered with the NCI Clinical Trial Reporting Program ID: NCI-2021–03,567 on June 16, 2021. The study can be found on clinicaltrials.gov, Identifier NCT04928599.

**Supplementary Information:**

The online version contains supplementary material available at 10.1186/s12913-022-08201-0.

## Introduction

The diagnosis of childhood cancer is unpredictable and rare, which precludes parents’ ability to plan for its financial repercussions. Children and their parents may come to diagnosis with characteristics that heighten risk for financial toxicity, that is, the harmful effects of the personal costs of cancer care [1, 2]. Direct expenditures and indirect losses (household income, work, and educational opportunities) as costs of care contribute to financial toxicity in three domains: (a) psychological responses, specifically, financial distress (worry about money), (b) new or worsened material hardships (food, housing, utility insecurity), and/or (c) detrimental financial coping behaviors (suboptimal treatment adherence that impacts care outcomes, debt non-payment that can lead to bankruptcy) [3–5]. Individual characteristics and social determinants of health both may contribute to worse financial distress among parents of children with cancer, similar to adults with cancer [6, 7]. For example, financial distress may be affected by gender-based role norms about providing financially for the family or caregiving for ill or elderly family members [4]. In pediatric cancer, this may mean that mothers and fathers experience childhood cancer-related financial distress and other domains of financial toxicity differently. Additionally, parents who are not fluent in English or have low literacy may encounter greater barriers to navigating programs that offer financial resources, or may lack social networks that provide tangible support to offset financial burden [8].

Both child and cancer characteristics may also impact the risk for financial toxicity. Acute lymphoblastic leukemia (ALL), the most prevalent type of cancer in children and curable in more than 90% of children, requires treatment that is intense and lengthy [9]. For example, most children with ALL require more than 2 years of cancer treatment to achieve cure but some treatment (such as intrathecal chemotherapy) can only be provided by specialized cancer treatment centers, thus incurring more costs for those who live distant from a treating center. Similarly, tumor genetics and disease response to therapy are used to guide risk-adapted therapy, such that children with high or very-high risk tumor genetic profiles or poorer response to initial therapy receive more intensive, hospital-based therapy than those with low-risk tumor genetic profiles or better response to initial therapy [10–12]. Beyond tumor genetics and initial disease response, patient race, ethnicity and socioeconomic status are independently associated with survival outcomes in ALL secondary to social determinants of health that may be amenable to care delivery and policy level interventions. (Gupta et al. ASH 2021, manuscript in preparation) [13–19].

Oral chemotherapy is the mainstay of the maintenance phase of ALL therapy [20]. Significant caregiving efforts are regularly required of parents to optimize their child’s adherence to oral medications, which—together with lengthy treatment and the associated financial burden—is a significant source of psychosocial stress [21, 22]. Newer targeted therapies, such as tyrosine kinase inhibitors or immunotherapy, [23] may substantially increase the cost of therapy for children with higher-risk ALL. The financial burden of accumulating treatment-related costs, along with declining household income due to treatment-associated parent work disruptions to provide caregiving, generates continual or cyclic financial distress [8].

**Fig. 1:**
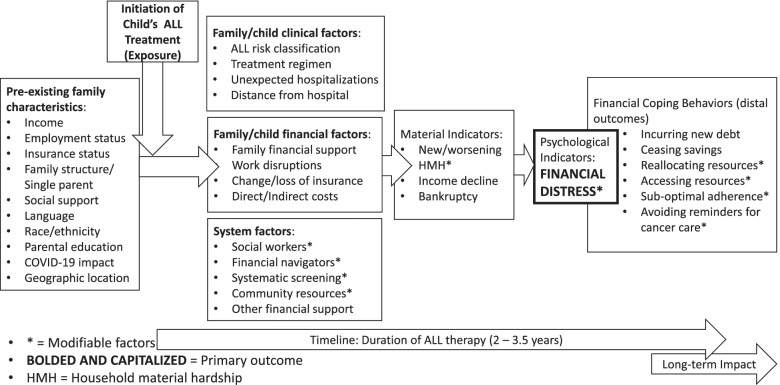
Model of financial toxicity during treatment for Acute Lymphoblastic Leukemia

The literature supports the existence of the three domains of financial toxicity in childhood cancer which are material hardship, financial distress and coping behaviors, as shown in the conceptual model that guides this study (Fig. 1) [8, 24]. However, no study has prospectively examined changes in material hardship, financial distress, and coping behaviors over time in this population. Our purpose is to identify, for parents and/or legal guardians of children (ages 1 to 14.9 years) newly diagnosed with ALL (“index child”), modifiable factors impacting the risk of financial distress and inflection points in this risk to inform future multi-level interventions to mitigate financial toxicity. Our aims are to:

1. Determine trajectories of financial distress.
2. Identify factors associated with financial distress over time. Candidates include socio-economic factors, clinical factors, indicators of material hardship, financial coping behaviors, and treating institution factors.
3. Explore parental experiences of financial distress, material hardship, and financial screening during a child’s ALL treatment.

Our study is based on the premise that financial distress, material hardship, and financial coping behaviors in the context of childhood ALL are amenable to multi-level intervention [25, 26]. Interventions informed by the data collected in this study are essential to reducing existing disparities in care delivery, health and survival outcomes for children diagnosed with ALL, and in quality of life for children and their family members. Our prospective, longitudinal study design will facilitate identification of critical inflection points for future interventions and leverages two existing National Cancer Institute (NCI)-funded infrastructures in the United States (US) to accomplish this aim: (a) the Children’s Oncology Group (COG), which prioritizes improving childhood cancer outcomes, and (b) the NCI Community Oncology Research Program (NCORP), which prioritizes improving the generalizability of research findings through collaborations with both Community and Minority/Underserved NCORP institutions across the US. This study will evaluate modifiable factors associated with financial distress and lay the groundwork for future multi-level interventions to improve financial distress during and after childhood cancer therapy [18, 27, 28]. Successful interventions will have implications for multiple down-stream consequences of financial distress. For example, parent factors that influence their engagement in system-level financial screening or connection to available resources will inform development and testing of interventions to support those behaviors and facilitate resource matching [18, 19]. Similarly, institutional factors that influence enactment of recommended regular, systematic screening for financial distress in pediatric oncology settings [29] will inform development and testing of systematic financial screening implementation strategies.

## Main text

### Methods

**Fig. 2:**
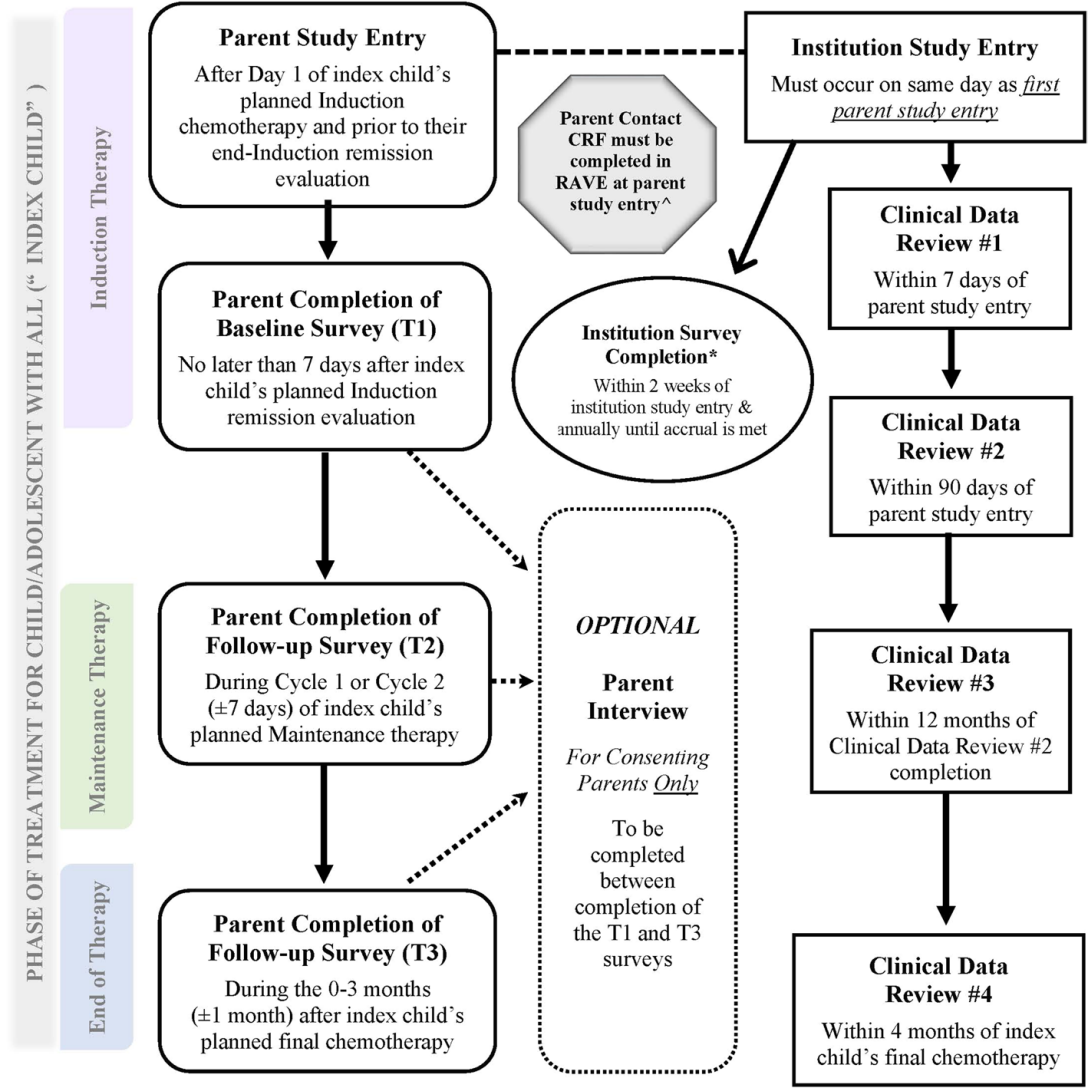
Study Design Schema for ACCL20N1CD: financial distress during treatment of acute lymphoblastic leukemia in the United States

Our study will use a prospective cohort design with an embedded optional qualitative study for a sub-cohort of participants. Parents participating in the cohort study will complete surveys at three time points (T) during the index child’s treatment—during induction therapy (T1), during maintenance therapy (T2), and around the time of completion of treatment (T3). For the sub-cohort of parents who agree to participate in the qualitative study and are selected, we will conduct an individual interview between T1 and T3. We will also ask research personnel at institutions that enroll at least one parent on the study to complete an annual survey of institution-level information. Details of the study design are shown in Fig. 2. The study is registered with the NCI (identifier NCI-2021–03,567) and was approved by the NCI Pediatric Central Institutional Review Board (CIRB).

### Setting

The study will be conducted at institutions in the NCORP network that participate in COG. NCORP institutions are geographically dispersed across the US, the District of Columbia, and US territories including Puerto Rico. They provide care and access to cancer clinical trials and care delivery research in community-based institutions and institutions with patient populations that include sizeable proportions of racial /ethnic minorities and other historically marginalized groups [30]. All 47 NCORP institutions that participate in COG may enroll eligible parents in this study.

### Participants

Participants will include parents or legal guardians (hereafter “parents”) of index children with newly diagnosed de novo ALL. The index child must be between the ages of 1 and 14.9 years at the time of their parent’s enrollment. Parents who speak English and/or Spanish are eligible if the parent has (a) the knowledge required to respond to the survey items, and (b) sole or shared responsibility for paying for the index child’s medical bills, other treatment-related expenses and basic material needs (clothes, food, housing). An index child may have more than one parent who meets eligibility criteria; in this situation, we will ask the parents to determine which one will enroll and complete the study assessments. This same parent must complete the study surveys at all three data collection time points plus the opt-in interview, if participating and selected. For index children with more than one household, we will ask the family to designate the primary household and, within that household, the parent who will complete all three surveys plus the opt-in interview, if selected.

### Study processes

Research personnel will approach potentially eligible parents after the first day of systemic Induction chemotherapy and prior to end-Induction remission evaluation (typically 4-week window). During the adult consent process, parents will be invited to participate in a future, optional interview and/or agree to be contacted about future research. Following written consent and prior to enrollment, research personnel will ask parents to complete a Parent Information/Contact Form which will include self-reported parent demographic data (parent date of birth, gender, race, ethnicity, and preferred language) to allow registration in the NCI’s enrollment system; this form will also capture the parent’s preferred contact day/time and mode of contact (telephone, text message, email). The research personnel at COG institutions will then register the parent in the NCI’s Clinical Trials Support Unit (CTSU) Oncology Patient Enrollment Network (OPEN) portal using these data, which will alert the study team to the need for survey distribution (see Data Collection). The study team will commence data collection when the parent has provided written consent, been verified as meeting eligibility requirements, and been registered in the CTSU OPEN system.

Pediatric CIRB waived assent from index children to extract a limited set of data about their disease and its treatment from the electronic health record (EHR). The justification is that parents consider their finances and financial worries sensitive matters which, other than in preparation for the responsibilities of adulthood, they prefer not to discuss with their children [31]. Assenting index children to EHR abstraction would require parents to discuss the study focus—harmful effects of the financial costs of the child’s cancer care—with their newly diagnosed child. Thus, parent willingness to participate in the study could be seriously constrained if child assent for data extraction were required*,* given that parents may be reluctant to burden their child with worries about the study topic.

#### Sampling

We will use convenience sampling procedures to accrue the quantitative cohort study, with the expectation that participating COG NCORP institutions will identify all eligible parents of children with newly diagnosed ALL and approach them about the study. Prior studies that have recruited through the NCORP have enrolled diverse samples [32, 33]. We will monitor the study distribution of accrued participant gender, race, and ethnicity on a monthly basis and communicate regularly with research personnel at participating institutions to understand, in real time, barriers to enrollment of parents who have been underrepresented in previous research of childhood cancer-related financial distress, including parents who are fathers, non-White, Hispanic/Latinx and/or prefer to complete some or all of the study requirements in Spanish. For the optional qualitative sub-study, we will purposively sample and interview parents from among those who opted in, until saturation is reached (i.e., we are no longer identifying new information or themes in the data). At least 15 parents will be included in the qualitative sub-cohort, including parents who prefer to be interviewed in Spanish.

#### Data collection

We will collect data from three sources: parents who enroll on study, the index child’s EHR, and institutions that enroll parents on study.

We will ask parents to complete a baseline (T1) and two follow-up (T2, T3) surveys over the course of the child’s treatment (respectively, during induction therapy, during maintenance therapy and then around the time of treatment completion). Parents of children whose ALL is refractory, relapses, or requires stem cell transplant or chimeric antigen receptor T-cell (CAR-T) will remain enrolled in the study if they have completed the T1 survey. These parents will be asked to complete one follow-up survey within 3 to 6 months of the child’s stem cell transplant or CAR-T therapy. Parents of children who die after the parent has completed the T1 survey will not be asked to complete follow-up survey(s) and will be removed from the study.

Parents will respond to the survey via a Research Electronic Data Capture (REDCap®) link. REDCap® is a web-based, Health Insurance Portability and Accountability Act (HIPAA)-compliant data collection platform, developed at Vanderbilt University and made available through the Clinical and Translation Science Award (CTSA) network. After the parent is enrolled within the enrollment portal and their Parent Information/Contact Form data are entered into the NCI’s Medidata Rave electronic data capture platform, we will use this information to send the parent a unique link to each REDCap® survey at the starts of T1, T2, and T3, using their indicated contact method preference (text message, email). If a parent does not submit a completed survey within 72 h, the link will expire, and we will provide a new link within the next 72 h. If a parent is unable to directly enter their survey responses into REDCap® within the specified time frame, research personnel at the enrolling institution will administer the survey by phone or an institution-approved remote platform. In this situation, the research personnel must contact the COG Research Coordinator to obtain a unique link to the REDCap® version of the survey for the appropriate time point. If neither of these options is possible, the parent may complete the survey using paper and pen; institutional research personnel will provide the parent with the survey for the particular data collection time point. As soon as the parent has completed the paper copy of survey, institutional research personnel will contact the COG Research Coordinator to obtain a unique link to the REDCap® version of the survey and then enter the parent’s responses into REDCap®.

We expect that parents can complete each survey in about 30 min, regardless of the data collection mode or time point. Parents may pause their work, if necessary, and return to complete and/or modify their responses prior to expiration of the link at 72 h. However, once the survey has been submitted in REDCap®, responses cannot be modified. The study team will request that institutional research personnel follow-up with a parent to ensure complete and unambiguous data, as needed. In rare cases, a member of the study committee may reach out to parents to obtain data that research personnel at the enrolling institution haven’t been able to collect. To reduce ambiguity around the meaning of missing data, we have included the response option “prefer not to answer” for survey items regarding particularly sensitive topics.

#### Study measures

Our study uses two types of measures: those to be completed by parents (parent survey, parent interview), and those to be completed by institutional research personnel (EHR extraction form, institutional survey).

##### Parent survey

We designed the parent survey to include items that assess the following components of our conceptual model (Fig. 1): (a) socio-economic status (SES), (b) financial distress, (c) material hardship, and (d) financial coping behaviors. *Indicators of parent SES* to be measured directly by survey items or derived from the data include: the parent’s social relationship to the index child (mother, father, other guardian), household income, household structure (household size, headed by single-parent or not), parent education level, parent primary language, parent health literacy level as measured using a single-item, [30] parent employment status (e.g., fulltime, part-time, not employed), parent employment benefits (e.g., paid time off, sick leave), parent change in employment status, the index child’s insurance type at diagnosis, census-derived income quintile for the primary residence[34] and distance/travel time to treating institution (derived using residence and institution zip codes). Baseline survey is provided in Additional file 1.

We will measure *financial distress* with the 8-item Personal Financial Wellness Scale (PFWS), previously known as the InCharge Financial Distress and Financial Wellness Scale [35]. The PFWS has been used in prior studies to describe financial distress in adults with newly diagnosed solid tumors [7]. Items ask for the perspective of the respondent as the financially responsible individual using a 10-point response scale. Item responses are totaled and then divided by 8. Mean scores can range from 1 to 10, with 1 indicating overwhelming financial distress and 10 indicating no financial distress. All eight PFWS items have been shown to load onto a single latent factor, [36] providing support for the scale’s uni-dimensionality.

For this study, the first and second authors engaged with three independent certified professional translators (two forward translations reconciled into one document, back translation of the reconciled forward translations), and the Inclusive Science Team at the second author’s NIH-funded CTSA (UL1TR002489; PI: J. Buse) in using a best practices [36] to obtain a valid linguistic translation and cultural adaptation of the PFWS for use by parents who prefer to use Spanish in completing study measures.

We will use 5 indicators to assess *material hardship*: (a) change in household material hardship (HMH), (b) change in parent-reported household income, (c) categorical change in percent federal poverty level (%FPL), (d) new consideration of or actual filing for bankruptcy, and (e) out-of-pocket expenses. Indicators of HMH include food, housing, transportation, and energy insecurities, which are amenable to interventions such as matching individuals with concrete resources (food assistance, travel supports) [19, 27, 37]. HMH is a proxy for poverty-exposures and, as a modifiable metric, may be a more useful indicator of SES than household income [37, 38]. We will use five items from the abbreviated form of the 13-item Oncology Economic Impact Study survey to assess food, housing, energy, and transportation insecurities [29]. HMH will be categorized by domain (endorsing food, housing, energy, or transportation insecurity) on a scale of 0 (no domains endorsed) to 4 (all four domains endorsed), and scored as a categorical variable (number of unmet material needs, range 0–4). We will collect *household income* as a continuous variable and use the data to calculate percentage change in income between time points. Catastrophic reduction in income related to medical expenses has been defined as a reduction exceeding 40%; more than 10% reduction can tip families into poverty [39, 40]. We will also examine household income and household size relative to the federal poverty level (FPL) for the data collection year, per the US Health and Human Services Poverty Guidelines [41]. Income poverty is defined as less than 100% FPL and low-income as less than 200% FPL, which is commonly used to determine eligibility for government programs such as public health insurance and food assistance. We will categorize % FPL as less than 100% FPL, between 100 and 200% FPL, between 200 and 300% FPL, and more than 300% FPL. We expect that about 20% of the index children and their parents will be living in poverty at diagnosis [42, 43]. We will use two items in the follow-up surveys (T2, T3) to assess new onset of consideration of or actual filing for bankruptcy. Similarly, we will use two items to assess direct (out-of-pocket expenses) and indirect (opportunities lost) costs. We will also explore direct and indirect costs in qualitative interviews with the participating study sub-cohort.

We will assess *financial coping behaviors* by asking parents to indicate how often (never, sometimes, often, or I don’t have this resource) they used 12 strategies to accommodate the costs associated with the child’s cancer care. The listed strategies include changes in health-related behaviors (forgoing recommended or needed health care for the index child or another family member) and changes in financial behaviors (accumulating credit card debt), similar to items used in prior studies of parents of children diagnosed with cancer [44]. We will also assess (yes, no, no-not eligible, no-I don’t know if I’m eligible) if the parent accessed financial resources, including from the government, the treating institution, and/or philanthropic organizations (Table 1).

**Table 1:** Modifiable secondary outcomes amendable to individual or healthcare delivery interventions

Aim	Measure	Objective	Analytic Plan	Modifiable target/future intervention
Aim 1	Personal Financial Wellness Scale	Determine trajectory of financial distress	Mixed model	Financial distress may be amenable to financial navigation (Shankaran, 2018) and other types of intervention
Aim 2	Personal Financial Wellness Scale	Identify socio-demographic, clinical, financial and institutional factors associated with financial distress over time	Mixed model	Predictors of financial distress will inform identification of at-risk groups to target with future interventions
Aim 2	Household income relative to the Federal Poverty Level (%FPL) Household Material Hardship (HMH) Bankruptcy Out-of-pocket costs	Describe treatment-related material hardship during treatment for pediatric ALL as assessed by 1) worsening or new HMH, 2) percent change in annual household income, 3) change in % FPL 4) consideration of or actual filing for bankruptcy and 5) parent-reported out-of-pocket costs	Descriptive statistics	Worsening HMH is amenable to resource-matching interventions (Garg, 2007 & 2015). %FPL and out-of-pocket costs may predict risk for and characterize HMH
Aim 2	Coping behaviors survey	Describe parent financial and health coping behaviors	Descriptive statistics	Parent coping behaviors such as cutting back on needed health care due to cost may be amenable to behavior change interventions Lack of information about available resources or not following through on referrals may be amenable to resource-matching and/or financial navigation
Aim 2	Institution Survey	Describe institutional financial resources and factors	Descriptive statistics	Implementation strategies may promote systematic financial screening and provision of referrals to available resources as needed

##### Parent interview

We will conduct semi-structured interviews with a sub-cohort of parents to enhance our understanding of financial distress, material hardship and financial coping behaviors among parents of children diagnosed with ALL. Study committee members trained in qualitative interviewing for research will conduct interviews by telephone or institution-approved remote platforms, per parent preference; a study committee member who is a bi-lingual (YMC), native Spanish speaker will interview parents who prefer to participate in Spanish. We expect interviews to last about 30 to 45 min, no matter the mode. The interview guide was informed by the conceptual model guiding the study (Fig. 1). Topics include: (a) distress related to financial costs, (b) how the parent approaches problem-solving and decision-making about financial coping behaviors, (c) whether someone has spoken with them about costs associated with ALL therapy and, if so, how the conversation went, and (d) what the parent would change for themselves or recommend to other parents regarding financial coping behaviors during childhood cancer therapy.

##### EHR extraction form

Participating sites will abstract clinically relevant information from the index child’s EHR at four time points. The first data will be entered at baseline and will include information from the Parent Information/Contact Form and the index child’s initial planned treatment regimen. Each subsequent clinical data abstraction form will guide the COG Research Coordinator in anticipated timing for subsequent surveys as well as inform sampling for the qualitative aim. The fourth and final clinical data abstraction form will classify the child’s final ALL risk classification, treatment regimen, and targeted healthcare utilization (emergency department visits, hospitalizations, and intensive care unit admission).

##### Institutional survey

Research personnel at each COG NCORP institution that enrolls at least one parent in this study will complete an annual survey about institution-level factors that may be associated with implementation of the recommendation for regular systematic financial distress screening of childhood cancer populations [29, 45]. The institutional survey includes items that ask about the availability of resources (social workers, financial advisors, navigators) for patients receiving care at the institution and their families. Survey items were adapted from a prior survey of pediatric oncology centers [46].

### Statistical considerations and analytic approach

#### Power calculation

We determined the sample size for aims 1 and 2 at 100, based on feasibility. Given the support for the reliability and validity of the PFWS provided by prior cross-sectional studies, we selected financial distress as measured by PFWS as our primary outcome. We expect to enroll parents in this study over a 2.5- to 3-year period, including a 6-month start-up period. Between 2010 and 2019, roughly 240 children per year diagnosed with ALL were enrolled onto treatment trials at the 47 COG institutions within the NCORP network. If half of these institutions activate this study, and 50% of eligible parents enroll, we estimate that 60 parents per year can be enrolled. Considering 10% attrition per year over 3 years, the study could enroll at least 100 parents within proposed accrual time. In a prior cross-sectional study of financial distress in cancer patients, [7] the PFWS mean score for patients under age 50 years was 5.0 (SD = 1.9); we expect that most parents who enroll in our proposed study will be young or middle-aged adults. In the prior study, for patients aged 50–64 years, the PFWS mean score was 5.7 (SD = 2.7) [7]. Assuming 100 parents enrolled and 73 complete the baseline survey plus one follow-up survey, we will have at least 90% power to detect a mean of paired differences of -0.5, with an estimated SD of paired differences of 1.3, and a significance level (alpha) of 0.050, using a two-sided paired t-test. We will have at least 80% power if the SD of paired differences is 1.5. These power calculations use estimates of SD for measurements at a single time point approximate to the results in Meeker et al. [7] not for differences in measurements from a baseline time point—these SDs are not readily available. We expect actual SDs of paired differences to be lower due to correlations between repeated observations, thus higher power in practice. A 0.5-point change in score is equivalent to 10% change in the mean 5.0 score from the Meeker study [7].

#### Analysis to address Aim 1: determine the trajectory of financial distress

We will compute PFWS scores using the established scoring algorithm [7, 35]. We will model continuous PFWS scores as a linear function of time; scores will be appropriately transformed to normality as necessary. We will fit a mixed model for mean PFWS score with an intercept and a main effect for time, as a categorical variable. We will use this model to examine the trend over time and estimate the slope of change (95% CI) in PFWS scores. We will consider parents with at least a baseline and an additional time point measurement (either T2 and/or T3) as evaluable. Research personnel will take steps in real time to ensure complete assessments and non-ambiguous responses. If missing data are unavoidable, we may explore alternative modeling assumptions.

#### Analysis to address Aim 2: identify factors associated with financial distress over time

We will use univariate mixed models to identify factors independently associated with the continuous PFWS score over time. We will employ multivariate mixed models and backward selection methods to identify factors jointly associated with the PFWS score; non-significant predictors will be excluded (if *p* >  = 0.05) to produce a final model. We will consider time (T1, T2, T3) as a 1 degree of freedom (df) variable in the model. Significant predictor interaction with time will be considered in the final multivariate model. We will evaluate and address multi-collinearity during model selection. Factors that we will consider include socio-economic variables, clinical variables, institutional variables, material hardship variables (annual household income, a continuous variable and HMH score, a categorical variable), and financial coping behaviors.

#### Analysis for exploratory aims

We will use descriptive statistics to report domains of financial distress informed by the conceptual framework. We will assess treatment-related material hardship as a) change in HMH score, b) percent change in annual household income, c) change in % FPL category, d) consideration of or actual filing for bankruptcy, and e) parent-reported out-of-pocket costs. We will assess potential coping behaviors by parent report of resources used and modification of financial or psychological behaviors. We will describe institutional factors associated with parent-reported financial distress, including the number of social workers, availability of philanthropic support (yes/no), and availability of financial advisor/navigation (yes/no). We will use institutional factor measurements via institution surveys obtained closest to the timing of the parent assessment.

#### Analysis to address Aim 3: explore parental experiences of financial distress, material hardship, and perceptions of financial screening during their child’s ALL treatment

We will secure verbatim transcripts of each audio-recorded interview in the source language, that is, in English for interviews conducted in English and in Spanish for interviews conducted in Spanish, the associated field notes and an interview summary. We will analyze these data using a directed content analysis approach and codebook of predetermined codes derived from our conceptual framework. Following a rigorous process and staying in the source language as much as possible to avoid losing meaning in translation, [47–50] two study team committee members trained in qualitative analysis will code each English language transcript, and two bi-lingual study committee members trained in qualitative analysis will code each Spanish-language transcript. These individuals will inductively code data segments that don’t fit predetermined codes by adding labels, definitions, and examples of data segments that reflect inductively derived codes to the codebook for use in future coding, then re-examining already coded data for examples of the new codes. Two bi-lingual individuals familiar with cultures of Spanish-speakers will work side-by-side to translate the concepts, categories and themes that emerged from the Spanish-language transcripts into English, with illustrative quotes in both the source language and English [50]. Study committee members trained in qualitative methods will use these translations in further analyses (e.g., comparing and contrasting concepts, categories and themes) of the entire qualitative data set. Once saturation is reached and no new themes are emerging from interviews conducted in Spanish or in English, our study team will agree on and synthesize the final themes. We will provide quotes in the source language in research reports of the work to address aim 3 [51].

### Participant retention and data quality considerations

We have a plan for close data monitoring and optimization of participant retention. We will identify missing parent survey data at the earliest possible time point, following the closure of the assessment window/expiration of survey link. We will run regular queries to identify missing assessments as well as missing data within the assessments. We will engage research personnel at the enrolling institution to assist us in determining the reason for missing assessments (e.g., illness, forgot, unable to make appointment). A study committee member will review completed surveys to identify missing items, and send reminders to parents and institutions via secure REDCap® link to encourage completion of missing items as soon as possible within the assessment window. If the T1 survey is missing, we will not include the parent in any analysis. However, if the T2 or T3 survey is missing, we will collect the reason for missing data with assistance from research personnel at the enrolling institution and may use the data as outlined in the statistical plan. We will also work closely through the NCORP network, including regular virtual meetings with COG research personnel at participating institutions, to discuss missing data and address challenges as proactively as possible. The NCORP network has been utilized in prior studies through COG and was successful in obtaining high-quality data.

## Discussion

From what is known at this point in time, no studies have prospectively examined trajectories of financial distress during ALL treatment. Our study aims to address this gap utilizing the existing robust research infrastructure of NCORP to enroll a diverse parent cohort based on language, geography, socioeconomic status, and self-identified race and ethnic categories to support the generalizability of our findings. Results of this investigation will be highly salient to a wide range of stakeholders given the potential associations between financial distress, treatment adherence, care outcomes and quality of life in pediatric ALL, the most common pediatric cancer. Upon study completion, we anticipate a robust understanding of how financial distress evolves during ALL therapy to inform rigorous studies in the future to accurately identify and intervene on this potentially catastrophic adverse effect of cancer care. We briefly discuss procedural, human subjects, and analytic considerations, and speculate about implications and future goals informed by this study.

### Procedural considerations

We recognize the time and resource commitment required for participation in clinical research. Though the participating institutions have infrastructure funded in part through the NCORP, observational or supportive care studies are reportedly not prioritized by institutions as often as treatment clinical trials [52, 53]. We therefore aim to operationalize a feasible and scientifically robust study to ensure collection of high-quality data by addressing multi-level factors known to contribute to study burden and thus negatively affect recruitment and retention of diverse samples in research of cancer populations [54, 55]. For example, the study will be conducted at NCORP institutions where racial/ethnic minorities and other under-served groups tend to seek care. Moreover, NCORP site research staff are trained in how to approach potential participants about studies for which they are eligible. By utilizing the REDCap® platform and other technologies, we expect to mitigate logistical challenges by (a) contacting parents directly using their preferred method and removing this burden from research staff at the enrolling institution, (b) letting parents complete the study surveys at their convenience and designing the surveys so they can pause/resume their work as needed, and (c) developing survey and data tracking procedures that use a multidimensional process to integrate data from the CTSU OPEN enrollment portal with data from REDCap® and Medidata Rave. We conducted a series of tests with the study committee in collaboration with parent stakeholders from the COG Patient Advocate Committee (PAC) to validate assumptions underlying our processes for data collection and minimization of missing data.

### Human subjects considerations

Because our study is designed to enroll parents, not children newly diagnosed with ALL, as the study participants, we partnered with our COG PAC representative (KA) and she accepted an invitation to join our study team. Together, we developed acceptable processes to identify, enroll, survey, and interview parents of children recently diagnosed with ALL, including those who prefer Spanish language, the second most common language in the US [56]. Further, the study consent document states that the parent’s data, including data from the semi-structured interview, will not be shared with members of the child’s healthcare team. We built data collection periods with as much leeway as possible given that parents can feel overwhelmed, experience consent fatigue, and may not have the bandwidth to complete surveys within narrow windows of time [57]. Because study time points are not tied to clinical questions, we believe these considerations are both reasonable and likely minimize participation burden for parents.

### Analytic considerations

Our primary aim is based on the hypothesis that financial distress changes linearly during a child’s treatment for ALL. If the results of the analysis to address this aim do not support this hypothesis, we will conduct exploratory contingency analyses. First, we will examine non-linear effects. Second, we will examine PFWS scores over time in stratified analysis by parent socio-economic factors (household income, insurance type) at T1. Finally, we will calculate descriptive statistics at each time point for individual items within the PFWS and explore its psychometric properties.

### Implication and future goals

As the cancer community and society in general comes to terms with the prevalence and long-lasting effects of financial toxicity for people affected by cancer, our study is timely. A conceptual framework derived from the literature informs our prospective, longitudinal data collection strategy using mixed methods to highlight inflection points for the testing of future interventions. These robust data will also inform the future intervention targets and components. This study assesses mechanisms that may contribute to financial distress, and utilizes qualitative data to enrich our understanding of acceptable procedures through which to develop and deliver future interventions from the perspective of the parents who directly experience the financial burden of their child’s cancer.

Finally, as highlighted in a recent New York Times opinion piece [58] by a parent whose infant daughter died of a brain tumor yet referred himself as “one of the lucky ones,” we urgently need policy and legislative level interventions to ease the financial burden of cancer care and its consequences for patients and their caregivers. We, meaning the pediatric oncology community, would be morally negligent to continue providing high-quality clinical care, curing nearly 90% of children diagnosed with ALL, while ignoring the costs that families incur throughout the illness trajectory and how these costs may contribute to well-described disparities in outcomes [59, 60]. Our hope is that this study and others in this research area will inform ongoing policy work, such as the Credit for Caring Act, [61] that includes legislation to provide financial support for families caring for ill family members, including children, or paid (rather than unpaid) family leave. Ultimately, multilevel approaches directed at individuals and multiple levels of the environment (interpersonal, institutional, healthcare system, social policy) are critical to mitigating financial toxicity. This study presents a key component of that multi-level approach. We thank parent participants in advance for contributing to these efforts.

## Supplementary Information

**Additional file 1.**

## Data Availability

No data have been captured to date for the research study. Surveys and other study materials are available upon reasonable request.

## Abbreviations

ALL: Acute lymphoblastic leukemia
CIRB: Central Institutional Review Board
COG: Children’s Oncology Group
CAR-T: Chimeric antigen receptor T-cell
CTSA: Clinical and Translation Science Award
CTSU: Clinical Trials Support Unit
EHR: Electronic health record
HIPAA: Health Insurance Portability and Accountability Act
HMH: Household material hardship
NCI: National Cancer Institute
NCORP: National Cancer Institute Community Oncology Research Program
OPEN: Oncology Patient Enrollment Network
PAC: Patient Advocate Committee
PFWS: Personal Financial Wellness Scale
REDCap®: Research Electronic Data Capture
SES: Socio-economic status
