# Supplementary material for: Rationale and design of Children’s Oncology Group (COG) study ACCL20N1CD: financial distress during treatment of acute lymphoblastic leukemia in the United States
Source: BMC Health Serv Res. 2022 Jun 28;22:832. doi: 10.1186/s12913-022-08201-0 (PMC9237978; doi:10.1186/s12913-022-08201-0)
Supplement: Supplementary file 1 — Additional file 1. [file 12913_2022_8201_MOESM1_ESM.pdf]

APPENDIX IV-A: PARENT BASELINE SURVEY (T1)

COG Parent Financial Study - Survey #1

Thank you for agreeing to participate in this study. Our goal is to find ways to better support families during a child's or adolescent's treatment for leukemia. While we hope that you will complete each item, if a specific question makes you feel uncomfortable, please select the option "prefer not to answer" when available, rather than leave it blank.

Personal/Demographic

Thank you once again for agreeing to participate in this study. First, we will ask a few questions about you.

1. **What is your relationship to the child receiving treatment for ALL ("your child")?**
   a. Mother
   b. Father
   c. Legal guardian (please specify relationship): ___________________
   d. Other (please specify relationship): ___________________

2. **What language do you speak at home most of the time?**
   a. English
   b. Spanish
   c. Other (please specify): ______________________

3. **In what language do you prefer to have medical conversations about your child?**
   a. English
   b. Spanish
   c. Other (please specify): ______________________

4. **What is the highest level of schooling that you completed?**
   a. Grade school or elementary school (Grades 1-8)
   b. Started high school but didn't graduate (Grades 9-12)
   c. High school diploma or GED
   d. Some college or special training after high school
   e. Associate's degree
   f. Bachelor's degree
   g. Graduate (Master's or doctoral degree)

5. **Please select the option that best describes your current marital status:**
   a. Married
   b. Living with a partner
   c. Single (living alone)
   d. Divorced or separated
   e. Widowed
   f. Other (please specify): _______________________

6. What is your current employment status?
   a. Full-time employed (you work an average of at least 30 hours per week)
   b. Part-time employed (you work an average of less than 30 hours per week)
   c. Unemployed
   d. Student (full-time student, not employed)
   e. Full-time homemaker
   f. Retired
   g. Other (please specify): ______________________

If you answered "Unemployed" for question 6, you may skip questions 7 and 8.

7. Please select the benefits you have through your work or school (select all that apply):
   a. Health insurance
   b. Paid time off
   c. Paid sick days
   d. Short-term disability
   e. Long-term disability
   f. I don't know or I'm not sure
   g. None of these

If you answered "I don't know or I'm not sure" or "None of these" for question 7, you may skip question 8.

8. Please select the benefits you have used since your child was diagnosed with leukemia (select all that apply):
   a. Paid time off
   b. Paid sick days
   c. Donated time from co-workers
   d. Short-term disability
   e. Long-term disability
   f. None; I have access to benefits but haven't used them

9. What type of insurance do you use to pay for your child with leukemia's healthcare? (Select all that apply; please include primary and secondary insurance.)
   a. Medicaid or state-funded low-income insurance program (e.g., CHIP)
   b. Private insurance (through work or school)
   c. Private insurance (purchased directly)
   d. Uninsured (self-pay)
   e. Other (please specify, including Military and insurance such as TriCare): _________________

10. How often do you need someone to help you read instructions or other written material from your or your child's health care providers or social workers?
   a. Never
   b. Rarely
   c. Sometimes
   d. Often
   e. Always

Now, we will ask some questions about your child.

11. Is your child Hispanic or Latinx?
   a. No
   b. Yes
   c. Prefer not to answer

12. What is your child's race?
   a. American Indian or Alaskan Native
   b. Asian
   c. Black or African American
   d. Native Hawaiian or Other Pacific Islander
   e. White
   f. Other (please specify): ________________________
   g. Prefer not to answer

13. Is there another parent or parent-like figure in your child's life?
   a. Yes
   b. No

If you answered "Yes" for question 13, please complete questions 14 through 16. If you answered "No," you may move on to question 17.

14. What is this person's relationship to your child?
   a. Mother
   b. Father
   c. Legal guardian
   d. Other (please specify relationship): ______________________

15. How old is this person?
   a. Age in years: __________
   b. Prefer not to answer

16. What is their highest level of education?

a. Grade school or elementary school (Grades 1-8)
b. Started high school but didn't graduate (Grades 9-12)
c. High school diploma or GED
d. Some college or specialized training after high school
e. Associate's degree
f. Bachelor's degree
g. Graduate degree (Master's or doctoral degree)

Household

This part of the survey asks about the household where your child mainly lived ***in the 6 months prior to their leukemia diagnosis****. Please answer these questions referring to the time* ***before*** *their diagnosis.*

17. What is your home address?

a. Street number and name: ____

____

City or town: ____

Zip code: ___________________

b. Prefer not to answer

18. In the 6 months prior to your child's diagnosis, how many people (including you and your child) were living in your household at least 50% of the time?

________ Number of people

________ Of these people, how many have an illness that requires significant medical care besides your child with leukemia (such as you or your partner, your parent(s) or another child)?

19. In the 6 months prior to your child's diagnosis, was there a time when you were not able to pay the rent or mortgage on time because of financial difficulties?

a. Yes
b. No
c. Does not apply
d. Not sure or don't know
e. Prefer not to answer

20. **In the 6 months prior to your child's diagnosis, how did you typically get to the hospital or clinic for your child's medical appointments?**
    a. Own car
    b. Rides from others
    c. Public transportation (bus, subway, train)
    d. Uber, Lyft, taxicab, etc.
    e. Medicaid transportation benefit
    f. Other mode (such as walking, etc.)
    g. Prefer not to answer

21. **In the 6 months prior to your child's diagnosis, did lack of reliable transportation ever keep anyone in your family from medical appointments, meetings, work or getting things needed for daily living?**
    a. Yes
    b. No
    c. Prefer not to answer

22. **In the 6 months prior to your child's diagnosis, did the gas, electric, oil or water company send you a letter threatening to shut off the gas, electricity, oil or water to your household for not paying bills?**
    a. Yes
    b. No
    c. Does not apply
    d. Not sure or don't know
    e. Prefer not to answer

The next set of questions ask about the food eaten in your household in the last 6 months, and whether you were able to afford the food you need. Please answer these questions thinking about the 6 months ***before*** *your child's diagnosis. For each statement below, please tell us whether the statement was* ***often true****,* ***sometimes true****, or* ***never true*** *for your household* ***in the 6 months prior to your child's diagnosis****.*

23. **"We worried whether our food would run out before we got money to buy more." Was that often, sometimes, or never true for your household in the 6 months prior to your child's diagnosis?**
    a. Often true
    b. Sometimes true
    c. Never true
    d. Not sure or don't know
    e. Prefer not to answer

24. **"The food that we bought just didn't last, and we didn't have money to get more." Was that <u>often</u>, <u>sometimes</u>, or <u>never</u> true for your household <u>in the 6 months prior to your child's diagnosis</u>?**
    a. Often true
    b. Sometimes true
    c. Never true
    d. Not sure or don't know
    e. Prefer not to answer

<u>Resources</u>

*The next set of questions are about the resources supporting your child. Please answer these questions thinking about your household **<u>in the 6 months prior to your child's diagnosis</u>**.*

25. **How many adults were financially supporting your child (this may include employed parents, guardians, and those paying child support)?**

    _______ (number)

26. **Please provide your <u>best estimate</u> of your household's total gross annual income (before taxes are withheld).**

    This includes any money earned from wages from anyone working (who contributes to your household's income), child support payments, rent from properties, social security, disability, veteran's benefits, unemployment benefits, or worker's compensation.

    $ ______________________

27. **<u>In the past month</u>, about how much did you and your family spend out-of-pocket for your child's medical care? <u>Include</u> out-of-pocket expenses for prescription drugs, copayments, and deductibles. <u>Do not include</u> health insurance premiums or expenses paid by your child's health insurance.**

    $ ______________________

28. **<u>In the past month</u>, about how much did you and your family spend out-of-pocket for other costs related to your child's care? <u>Include</u> out-of-pocket expenses for transportation, childcare (for your child with leukemia or other children in the home), meals, housing upkeep, and homeschooling.**

    $ ______________________

*Many families are able to find financial support from existing resources. Please tell us if your family is **currently** receiving any of the following supports?*

29. **Medicaid health insurance for your child**
    a. Yes
    b. No, my child is not eligible
    c. No, my child is eligible, but we decided not to apply
    d. No, I do not know if my child is eligible

30. **Food stamps (e.g., SNAP)**
    a. Yes
    b. No, I am not eligible
    c. No, I am eligible but decided not to apply
    d. No, I do not know if I am eligible

31. **Free school breakfast or lunch**
    a. Yes
    b. No, my child(ren) are not eligible
    c. No, my child(ren) are eligible but we decided not to apply
    d. No, I do not know if my child(ren) are eligible
    e. Does not apply, my child(ren) are not in school

32. **Food from a food pantry (sometimes called a food bank or soup kitchen)**
    a. Yes
    b. No

33. **Energy assistance (e.g., discounted gas or electric bills, such as LIHEAP or utility discounts)**
    a. Yes
    b. No, I am not eligible
    c. No, I am eligible but decided not to apply
    d. No, I do not know if I am eligible

34. **Disability benefits (e.g., Supplemental Security Income or "SSI")**
    a. Yes
    b. No, I am not eligible
    c. No, I am eligible but decided not to apply
    d. No, I do not know if I am eligible

35. **Unemployment benefits**
    a. Yes, and I have received my check(s)
    b. Yes, but I have not received a check yet
    c. No, I am not eligible
    d. No, I am eligible but decided not to apply
    e. No, I do not know if I am eligible

36. Free government cell phone (e.g., Obama phone)
   a. Yes
   b. No, I am not eligible
   c. No, I am eligible but decided not to apply
   d. No, I do not know if I am eligible

37. Low-income or subsidized housing (sometimes called Section 8 housing)
   a. Yes
   b. No, I am not eligible
   c. No, I am eligible but decided not to apply
   d. No, I do not know if I am eligible

Some families receive support from their treating institution or hospital. Please let us know if you have received any of the following support.

38. Needed medical care for your child at reduced cost
   a. Yes
   b. No, I am not eligible
   c. No, I am eligible but decided not to apply
   d. No, I do not know if I am eligible

39. Financial support from your child's treating institution (this may include travel vouchers or gift cards to cover costs such as groceries or bills)
   a. Yes
   b. No, I did not need this support
   c. No, I needed this support but did not ask

Now we would like to ask a few questions about how satisfied you are with the support you receive from other people in your life (such as family, friends, community, parents like you). Please select one of the three response options for each type of support.

40. In general, are you satisfied with the amount of emotional support related to your child's illness and care that you have received from others?
   a. Not satisfied
   b. Satisfied
   c. Very satisfied

41. In general, are you satisfied with the amount of practical, day-to-day help related to your child's illness and care that you have received from others?
   a. Not satisfied
   b. Satisfied
   c. Very satisfied

42. In general, are you satisfied with the amount of information or advice related to your child's illness and care that you have received from others?
a. Not satisfied
b. Satisfied
c. Very satisfied

<u>Coping</u>

As a result of leukemia-related changes in household expenses and parent employment, have you had to do any of the following ***<u>since your child's diagnosis with leukemia</u>****?*

		Never	Sometimes	Often	I don't have this resource
43.	Dip into your savings				
44.	Dip into retirement accounts				
45.	Take out a loan or increase your level of credit card debt				
46.	Take a new job, a different job, or increase hours to increase your income				
47.	Not take another job or not pursue a new opportunity because you were concerned about losing current income or health insurance coverage, or both				
48.	Initiate or receive money from fundraiser or crowd-funding (e.g., GoFundMe)				
49.	Receive money from a family member or friend				
50.	Cut back on contributions to retirement or education accounts				
51.	Move to a new home to reduce mortgage or rent				
52.	Cut back on spending for vacations, travel or relaxation				
53.	Cut back on spending for afterschool or summer activities for your children				
54.	Cut back on spending for family birthday and holiday celebrations				

		Never	Sometimes	Often	I don't have this resource
55.	**Cut back on spending for groceries**				
56.	**Cut back on spending for medical care, dental care, or mental health or psychological care**<br><br>**If "sometimes" or "often," for which type of care? (Please select all that apply.)**<br>a. Medical care<br>b. Dental care<br>c. Mental health or psychological care				
57.	**Cut back on spending for necessities for your family members, such as clothing, transportation, or home utilities**				
58.	**Cut back on spending for education**<br><br>**If "sometimes" or "often," who was this for? (Please select all that apply.)**<br>a. Your child with leukemia<br>b. Yourself or other adult(s) in your family<br>c. Another child in your family				
59.	**Delay or skip filling prescription medications due to the cost of medication**<br><br>**If "sometimes" or "often," who was this for? (Please select all that apply.)**<br>a. Your child with leukemia<br>b. Yourself<br>c. Someone else in your family				
60.	**Make existing medications last longer by skipping or reducing doses due to the cost of medication**<br><br>**If "sometimes" or "often," who was this for? (Please select all that apply.)**<br>a. Your child with leukemia<br>b. Yourself<br>c. Someone else in your family				

		Never	Sometimes	Often	I don't have this resource
**61.**	**Miss appointments due to concerns about cost**<br><br>**If "sometimes" or "often," who was this for? (Please select all that apply.)**<br>a. Your child with leukemia<br>b. Yourself<br>c. Someone else in your family				

62. Are there any other changes in how you manage your finances that you would like to tell us about?

____

____

____

Financial Distress/Wellness

[This last section includes the Personal Financial Wellness Scale, previously InCharge Financial Distress/Financial Well-Being Scale. This scale is an 8-item measure and has been previously published: Prawitz, Aimee and Garman, E. Thomas and Sorhaindo, Benoit and O'Neill, Barbara and Kim, Jinhee and Drentea, Patricia, Incharge Financial Distress/Financial Well-Being Scale: Development, Administration, and Score Interpretation (2006). Journal of Financial Counseling and Planning, Vol. 17, No. 1, 2006, Available at SSRN: https://ssrn.com/abstract=2239338]

Thank you for completing the first survey for this study! We appreciate your time and responses. You will receive your next survey between 6 and 9 months from now. Please reach out us with any questions, COGParentFinancialStudy@childrensoncologygroup.org.
